# Open science versus commercialization: a modern research conflict?

**DOI:** 10.1186/gm316

**Published:** 2012-02-27

**Authors:** Timothy Caulfield, Shawn HE Harmon, Yann Joly

**Affiliations:** 1 Health Law and Science Policy Group, Law Centre, University of Alberta, Edmonton, T6G 2H5, Canada; 2 School of Law, University of Edinburgh, Old College, South Bridge, Edinburgh, EH8 9YL, UK; 3 Centre of Genomics and Policy, Faculty of Medicine, Department of Human Genetics, McGill University, 740 Dr. Penfield Avenue Suite 5200, Montreal, H3A 1A4, Canada

## Abstract

**Background:**

Efforts to improve research outcomes have resulted in genomic researchers being confronted with complex and seemingly contradictory instructions about how to perform their tasks. Over the past decade, there has been increasing pressure on university researchers to commercialize their work. Concurrently, they are encouraged to collaborate, share data and disseminate new knowledge quickly (that is, to adopt an open science model) in order to foster scientific progress, meet humanitarian goals, and to maximize the impact of their research.

**Discussion:**

We present selected guidelines from three countries (Canada, United States, and United Kingdom) situated at the forefront of genomics to illustrate this potential policy conflict. Examining the innovation ecosystem and the messages conveyed by the different policies surveyed, we further investigate the inconsistencies between open science and commercialization policies.

**Summary:**

Commercialization and open science are not necessarily irreconcilable and could instead be envisioned as complementary elements of a more holistic innovation framework. Given the exploratory nature of our study, we wish to point out the need to gather additional evidence on the coexistence of open science and commercialization policies and on its impact, both positive and negative, on genomics academic research.

## Background

Article 27 of the Universal Declaration of Human Rights (1949), Article 15 of the International Covenant on Economic, Social and Cultural Rights (1966), and Article 15 of the UNESCO Universal Declaration on Bioethics and Human Rights (2005) all articulate the obligation to share scientific knowledge and the right to share in the benefits of scientific knowledge. But like the concept of 'benefit sharing', the idea of optimally sharing and maximally utilising scientific knowledge is fraught with complexity, confusion, and policies at seemingly stark cross-purposes. The matter is further complicated by the fact that research is becoming more expensive, research structures are growing more complex and fractured, and innovation levels, at least in the pharmaceutical setting, are not increasing [1].

Efforts to meet these legally mandated needs and to simultaneously improve research outcomes have resulted in researchers being confronted with complex and seemingly contradictory instructions about how to perform their tasks. This is particularly vexing for university-based genomics researchers. On the one hand, they are told to commercialize their research by patenting, licensing, and forming close partnerships with industry, which has particular skills, financial assets to facilitate the translation of knowledge into products, and objectives. It is presumed that this will generate maximum wealth and benefit through the quicker introduction of socially useful knowledge and products. On the other hand, researchers are encouraged to share data and disseminate knowledge quickly (that is, to adopt an open science model) so as to foster scientific progress, meet humanitarian goals, and (again) maximize the impact of research.

As genomics research leaders with mature regulatory frameworks and enviable regulatory capacity, and as regular expositors of human rights rhetoric, Canada, the United Kingdom (UK), and the United States (US) might be expected to take a lead in clarifying the message to researchers and rationalizing the expectations to which they are held. While we recognize that other jurisdictions will have an equally strong interest in this regard (for example, Japan and the BRIC countries - Brazil, Russia, India and China), space limits our ability to address them, and, in any event, this paper is not intended as a comprehensive survey/study. Below we discuss the prevalence of these conflicting messages in Canada, the UK, the US, and internationally, and highlight some of the practical difficulties they are creating within the global research community. In doing so, we focus on the bioscience sector, which has been the subject of significant expectation and support, and we offer some hope to researchers that a rational course is not impossible.

## Discussion

### Commercialize and translate

While there are numerous social and political forces at work in generating the above-noted pressure to commercialize research, two in particular are noteworthy. The first relates to the need, recognized by governments and imposed on researchers, to secure research funds from sources other than government [2-5]. The second, and arguably more important, force is the desire to position university research as an engine of economic growth. The pressure to 'translate' has grown sharper since the addition of a 'third role' for universities in the 1990s; to 'knowledge producer' and 'knowledge transmitter' was added 'economic developer' [6].

This paradigm shift has resulted in increasing expectations that university researchers will build ties with industry and produce research with commercial potential. In the biotechnology sector, for example, knowledge-driven biotech start-up clusters associated with universities were prompted [7]. By 2003, the high technology cluster in Oxfordshire, England contained some 1,400 firms and employed some 37,000 people [8]. Pursuing this partnering agenda has demanded that universities overcome existing cultural values - many of which contributed to the excellence for which they became known in the first place - and that they adopt a more entrepreneurial approach to their activities [9]. While the commercialization of academic research has generated mixed results at best [10,11], the imperative to commercialize university research persists, and can be found throughout the world in both soft 'nudges' (expressed in policy statements, university technology transfer office (TTO) mandates and practices, funding opportunities, and intellectual property (IP) 'toolkits' available to researchers), and hard obligations (expressed in research networks and public funding agency requirements and agreements). Importantly, as highlighted in Table 1 this pressure is more than mere political posturing or bureaucratic musing. The idea that researchers should commercialize their work is part of the official mandate of public funding agencies and research networks, as demonstrated in the review below.

**Table 1:** Policies and guidelines encouraging commercialization

Canadian Institutes of Health Research (CIHR), *Canadian Institutes of Health Research Act *(2000)	The objective of the CIHR is to excel, according to internationally accepted standards of scientific excellence, in the creation of new knowledge and its translation into improved health for Canadians, more effective health services and products and a strengthened Canadian health care system, by ... encouraging innovation, facilitating the commercialization of health research in Canada and promoting economic development through health research in Canada
Genome Canada, *Guidelines for Funding Large-scale Genomics Research Projects *(2010)	Appendix A - Evaluation Criteria ... B. Benefits for Canada 2. The demonstration of how anticipated results will contribute to job creation, economic growth, development of a product, service, licenses or the creation of start-ups 3. The effectiveness of the proposed plans for commercialization, technology transfer and the handling of intellectual property (where applicable)... 4. The potential for use or commercialization (where appropriate) of the anticipated results and the extent to which the proposed research will further the development of new methods, perspectives and/or technology
Stem Cell Network (Canada), *Commercialization *strategy (2011)	The Network's commercialization strategy is driven by its mandate to support the commercialization of its funded research, and by a desire to act more broadly as a catalyst for commercialization across the whole Canadian stem cell research sector
California Institute for Regenerative Medicine (CIRM; US), *CIRM Stem Cell Grant Regulations *(2009)	...a Grantee shall make reasonable efforts to develop, commercialize or otherwise bring to practical application CIRM-funded technology or CIRM-funded inventions
Howard Hughes Medical Institute (HHMI; US), *Intellectual Property and Licensing Policies - Licensing by Host Institutions to Companies *(2008)	...the host institution is responsible for efforts to commercialize intellectual property developed in HHMI laboratories at the site
Howard Hughes Medical Institute (US), *Intellectual Property Policy *(2007)	The host institution's technology transfer office takes the lead on licensing or other commercialization activities with respect to subject property developed at the site
Department of Health (UK), *Research Governance Framework for Health and Social Care*, 2d edition (2005)	Consideration must be given to the exploitation of intellectual property rights
Medical Research Council (UK), *Good Research Practice *(2005)	MRC-funded researchers are expected to maximize the prospects of research being taken into practice through the commercial route by protecting intellectual property rights
Medical Research Council (MRC; UK), *Translational Research Strategy *(2008)	The MRC recognizes the important role collaborations can play in helping to meet our priorities of translating research into healthcare improvements and enhancing economic prosperity. As such, the MRC welcomes applications involving academic/industry collaborations...
Biotechnology and Biomedical Sciences Research Council (BBSRC; UK), *Strategic Plan *(2010)	BBSRC seeks to understand the most critical bioscience challenges facing industry and create opportunities for engagement between academia and industry. This ensures that the research and training we fund promote innovation and generate impact. We will increase the range and depth of our interactions with business ...

In Canada, the National Research Council has adopted a strategy that 'downplays basic research' and emphasizes work that will 'attract industry partners and generate revenue' [12], a shift that has been reproduced at the provincial level (for example, see the Alberta experience [13]). The commercialization objective has even been explicitly built into the enabling legislation of the Canadian Institutes of Health Research (CIHR). One of the legislated objectives of CIHR is 'facilitating the commercialization of health research in Canada and promoting economic development through health research in Canada' [14]. The Canadian Networks of Centres of Excellence Program, developed in 1989 to mobilize Canada's best research teams, has been developed around the idea of altering the academic culture and managing scientific research to make it more commercial [15]. In its recent 20th anniversary report, the Canadian Networks of Centres of Excellence stated that it would focus in the coming years on increasing industrial investment in research and development partnership and accelerating the commercialization of technologies, products and services [16].

This emphasis is recreated at the science-specific level. For example, the Canadian Stem Cell Network is mandated to support the commercialization of its funded research; since 2005, it has aligned its research program to conform to the three main routes to market (cells, drugs, and tools), and has erected funds and produced practical advice to facilitate commercialization [17]. In 2010, Genome Canada, an agency that supports large-scale genomic and proteomic projects, launched a pilot program called Entrepreneurship Education in Genomics to facilitate education of the Canadian genomics research community on how to create and capture value from their research and translate their discoveries into marketable applications, products, technologies, systems and processes [18]. This can be seen as an application of its *Guidelines for Funding Large-scale Genomics Research Projects *[19], which include effectiveness evaluations of commercialization plans for funding genomics projects.

As in Canada, numerous UK government policies and funding programs have been aimed at encouraging and improving university-to-business technology transfer, including the University Challenge and HEROBC [20,21]. In October 2011, the UK government announced a £220 million program to translate Britain's research strengths, including those around stem cell research, into manufacturing success by facilitating academia-industry partnering through joint centers of excellence [22]. This notion of university-industry collaboration and commercialization of outputs has spilled over into funding agency policies. For example, the Medical Research Council (MRC) *Good Research Practice *policy articulates an economic mission and stipulates that MRC-funded researchers are expected to maximize the prospects of research being taken into practice through the commercial route by protecting IP rights [23]. This policy also states that traditionally commercial and knowledge-enclosing material transfer agreements and confidentiality agreements are important for protecting resources that may have value. Similarly, the MRC's *Operational and Ethical Guidelines for Human Tissue and Biological Samples for Use in Research *[24] state that the MRC's mission is to support research that will ultimately benefit human health and that commercial involvement is crucial to this effort. Thus, access by the commercial sector to samples of human material collected in the course of MRC-funded research should be facilitated. Finally, under its *Translational Research Strategy *[25], the MRC acknowledges 'the important role collaborations can play in helping to meet our priorities of translating research into healthcare improvements and enhancing economic prosperity', and it 'welcomes applications involving academic/industry collaborations.'

In its *Strategic Plan *[26], the UK's Biotechnology and Biomedical Sciences Research Council (BBSRC) articulates three 'enabling themes': (1) knowledge exchange, innovation and skills; (2) exploiting new ways of working; and (3) partnerships. Regarding the first theme, the BBSRC explains:

BBSRC, in partnership with other funders, will develop a better understanding of the various routes and barriers to translation in different sectors. We will seek to deliver innovative solutions, focusing not only on intellectual property but more broadly on intellectual assets. We will also increase support for people in translational roles, and develop enterprise skills in researchers. ... BBSRC sustains a high quality research base that supports innovation in important UK business sectors including agriculture, food and drink, pharmaceuticals, healthcare, chemicals and biotechnology. Discovery and production activities in these industries depend on scientific advances in the academic community. BBSRC seeks to understand the most critical bioscience challenges facing industry and create opportunities for engagement between academia and industry. This ensures that the research and training we fund promote innovation and generate impact. We will increase the range and depth of our interactions with business...

These commercialization policies are not limited to universities and their primary funders, but are also advanced by other traditionally solidarity-based institutions. For example, the UK Department of Health, in its *Research Governance Framework for Health and Social Care *[27], states: 'Consideration must be given to the exploitation of intellectual property rights.' It has also identified the NHS as an important contributor to innovation capable of capturing new technologies and ensuring that those inventions that can make more income available for improving the health service are appropriately developed and exploited.

In the US, which has a long history of commercializing university research [28,29], the heightened pressure to work with industry is perhaps best exemplified by President Obama's 2011 State of the Union address where he unveiled his 'Startup America' initiative [30] and called upon the research community to help stimulate the economy [31]. He characterized this wealth- and job-creation plea as 'our generation's Sputnik moment' [31,32]. President Obama's initiative is multi-faceted but seeks to boost overall innovation and entrepreneurship in America by encouraging private investment in startups. One part of this initiative is the National Science Foundation's Innovation Corps (I-Corps) program, which, through public-private partnerships and funding opportunities, will 'guide promising research with commercial potential out of university laboratories' of current or previously funded National Science Foundation grantees [33]. Although adopting a more nuanced perspective on commercialization, a recent report from the National Research Council Committee on management of university IP nevertheless concluded that 'the goal of expeditious and wide dissemination of discoveries and inventions places intellectual property based technology transfer squarely within the research university's core missions of discovery, learning, and the promotion of social well-being' [34]. Finally, as in Canada, the commercialization imperative is reproduced at the state level (for example, see the California Institute for Regenerative Medicine [35]), and at the private philanthropic level (for example, see the Howard Hughes Medical Institute [36]).

Importantly, empirical research has confirmed the mounting intensity of this commercialization pressure. For example, a recent study of the perceptions of Canadian TTOs found that, '[w]ithout exception, all technology transfer professionals interviewed believed that commercialization pressure has increased over the past 10-15 years' [37]. As noted by one TTO respondent when asked if commercialization expectations have intensified: 'Very much so, dramatic. The pressure is coming from the government, the federal government and provincial government.' So not only are pressures mounting, but researchers are becoming increasingly sensitive to those pressures. Studies of the Australian [38] and Canadian genomics and stem cell research communities (both of researchers and the public) have also illustrated some of the challenges and frustrations created by the increasing commercialization of academic research [39-41], including the disconnection with the fundamental research reality, negative impact of commercial secrecy on academic research, and ambivalence of researchers toward gene patents.

### Open scientific collaboration

Despite the above policies, and perhaps following the mixed results achieved under them, there is also a movement, under a seemingly opposing ethos, to openly share scientific knowledge and to forge a model of 'open scientific collaboration'. Though removed in many ways from the open access and open innovation models that transformed the information and communication and the business sectors [42,43], it reflects them in its ambition to create a more open, collegial, and collaborative research environment, and it has been advanced over the past decade by several moves in a wide array of disciplines [44,45]. A common broad justification for open scientific collaboration (and increased sharing) is the idea, expressed succinctly by the BBSRC, that making data more readily available will reinforce open scientific enquiry and stimulate new investigations and analyses, and efforts at doing so should be led by the scientific community to ensure (1) data quality, (2) the formation of good practice, and (3) the natural embedding of sharing into the scientific culture [26].

In the bioscience sectors, this model has been pushed by leading international organizations and the research community itself [46-48]. UNESCO's *Universal Declaration on the Human Genome & Human Rights *[49], which is aimed at member states and research stakeholders, emphasizes the right of scientific inquiry within a human rights framework and subject to dignity-based limitations (Articles 10 to 15). It states that benefits should be shared (Article 12), articulates the duty to present and utilize findings (Article 13), and directs states and organizations to cooperate and exchange scientific knowledge and information (Article 19). UNESCO's *International Declaration on Human Genetic Data *[50] aims to foster international medical, scientific and cultural cooperation and fair access, and international dissemination of scientific knowledge, particularly between industrialized and developing countries (Article 18). It directs researchers to establish cooperative relationships based on mutual respect, and to circulate data so as to foster knowledge-sharing. The International Organisation of the Human Genome (HUGO), which considers databases and genetic data to be global public goods, has also been a proponent of open data sharing in its various ethics policies [51].

Similarly, the Organisation for Economic Co-operation and Development (OECD), in its *Principles and Guidelines for Access to Research Data from Public Funding *[52], states that the value of data lies in their use. Most importantly, it states that full and open access to scientific data should be adopted as the international norm associated with publicly funded research, so that expensive and unnecessary duplication of data collection can be avoided and the overall efficiency of publicly funded research can be improved. While these guidelines note the importance of protecting the rights and interests of all stakeholders, and concede that access to data might be limited on certain grounds (including national security, trade secrets, conservation, legal process, and privacy and confidentiality), they approach these limitations with a view towards preserving as much sharing potential as possible.

Strangely, given the commercialization positions already identified above, some of the strongest open science statements have been proclaimed by the research funding agencies themselves. For example, Genome Canada has committed itself to the principle of rapid sharing of its financed research projects and unique resources. Its *Data Release and Resource Sharing Policy *[53] and its *Policy on Access to Research Publications *[54] convey the key elements of this position to grant beneficiaries. Moreover, Genome Canada will review the beneficiaries' proposed data and resource sharing plan to verify that it conforms to Genome Canada policies and that funds will not flow until an acceptable plan has been approved and incorporated into the terms of award. Somewhat in paradox with its commercialization mandate, CIHR expresses in its *Policy on Access to Research Outputs *[55] its 'fundamental interest in ensuring that the findings that result from the research it funds, including research publications and publication-related data, are available to the widest possible audience, and at the earliest possible opportunity.'

In the UK, the MRC *Data Sharing Policy *[56] characterizes publicly funded research data as a public good and stipulates that restrictions imposed on access to publicly funded data should be limited. Data should be made available to the maximum extent possible, and should be shared in a timely and responsible manner. As such, the MRC has made data-sharing a prerequisite to funding, and has directed that data-sharers receive full and appropriate recognition. The BBSRC *Data Sharing Policy *[57] opines that bioscience datasets are important to the wider scientific community, and that re-use of data can lead to new scientific understanding. Thus, it offers substantive support for managing and sharing data and it instructs other funders, academic institutions, and new users to recognize sharing efforts. The BBSRC policy elaborates on data-sharing methods and instructs researchers to retain the data in accessible formats for a period of 10 years after project completion, in keeping with BBSRC guidance on good scientific practice. Likewise, the Wellcome Trust *Policy on Data Management and Sharing *[58] stipulates that it expects researchers to maximize the availability of data with as few restrictions as possible, and it articulates the belief that success in maximizing the value of research depends on fostering a culture in which both data generators and data users act with integrity and transparency in managing, using, and sharing data. To foster this, it states that all data users must acknowledge the sources of their data, and must work cooperatively to: (1) ensure that datasets are developed and maintained for use by the research community; (2) recognize the contributions of researchers who generate, preserve, and share datasets; and (3) develop best practice for data sharing in different fields, recognizing that different data types raise distinct issues and challenges.

The Wellcome Trust requires applicants to submit a data management and sharing plan whenever research is likely to generate data that will hold significant value as a resource for the wider research community.

In the US, the National Institutes of Health (NIH), in its *Final Statement on Sharing Research Data *[59], explains the Institutes' position to the effect that 'data sharing is essential for expedited translation of research results into knowledge, products, and procedures to improve human health.' More interesting, the statement requires that applicants seeking $500,000 or more in direct costs in any year of the project period must include a data-sharing plan in their application, stating how they will share the data or, if they cannot share the data, why not. The *Global Health Data Access Principles *of the Bill & Melinda Gates Foundation has adopted a similar approach, stipulating that, when possible, data should be deposited in public-access repositories. If this is not possible, prospective grantees should propose alternatives for access, with consideration given to the ease of discovery of the existence of the data set and maintenance of long-term access [60]. The Howard Hughes Medical Institute, while expecting an investigator's host institution to commercialize research outputs, also has a *Sharing of Publication-related Materials, Data and Software *policy [61], which states its mission supports broad dissemination of research tools, and requires databases that are too large to be included in a publication (but which are nonetheless integral to the publication) be made freely available by other means (for example, on-line at no cost, with no restriction on research use, and in a highly accessible manner [61]).

This open scientific collaboration philosophy has resulted in the creation of a range of open access journals, publicly accessible biobanks, and more. Recently, it has led a large group of major health research funders to issue a *Joint Statement of Purpose *[62], which sets out their belief that making datasets available to those beyond the original collectors in a timely and responsible manner will generate (1) faster progress in improving health, (2) better value for money, and (3) higher quality science. The signatories have agreed to work jointly and to call on other actors, including governments, to adopt data-access policies that promote sharing and use. The statement is founded on the principles of equity, ethics, and efficiency, and goes on to outline immediate goals and longer-term aspirations, which is important in a climate where academic researchers have been encouraged to seek commercial opportunities and IP rights over their inventions [63]. A sampling of open science research policies is offered in Table 2.

**Table 2:** Policies and guidelines promoting open science

*Universal Declaration on the Human Genome and Human Rights *(1997)	Article 12(a): Benefits from advances in biology, genetics and medicine, concerning the human genome, shall be made available to all, with due regard for the dignity and human rights of each individual
*International Declaration on Human Genetic Data *(2003)	Article 18(c): Researchers should endeavour to establish cooperative relationships, based on mutual respect with regard to scientific and ethical matters and, subject to the provisions of Article 14, should encourage the free circulation of human genetic data and human proteomic data in order to foster the sharing of scientific knowledge, provided that the principles set out in this Declaration are observed by the parties concerned. To this end, they should also endeavour to publish in due course the results of their research
HUGO, *Statement on Human Genomic Databases *(2002)	Human genomic databases are global public goods. ... The free flow of data...should be encouraged
*Bermuda Principles *(1996)	...all human genomic sequence information, generated by centres funded for large-scale human sequencing, should be freely available and in the public domain in order to encourage research and development and to maximise its benefit to society
*Fort Lauderdale Agreement *(2003)	Rapid release of DNA sequence data should be extended to all sequence data. Rapid pre-publication release should apply to other types of data from other large-scale production centers specially established as 'community resource projects'
*Toronto Statement *(2009)	...attendees endorsed the value of rapid pre-publication data release for large reference datasets in biology and medicine that have broad utility and agreed that pre-publication data release should go beyond genomics and proteomics studies to other datasets - including chemical structure, metabolomic, and RNAi datasets, and annotated clinical resources (cohorts, tissue banks, and case-control studies)
*Joint Funders Statement *(2011)	Goals: ... *Data sharing is recognized as a professional achievement* Funders and employers of researchers recognize data management and sharing of well-managed datasets as an important professional indicator of success in research *Well documented data sets are available for secondary analysis* Data collected for health research are made available to the scientific community for analysis which adds value to existing knowledge and which leads to improvements in health
Organisation for Economic Cooperation and Development (OECD), *Principles and Guidelines for Access to Research Data from Public Funding *(2007)	Full and open access to scientific data should be adopted as the international norm associated with publicly funded research
Canadian Institutes of Health Research, *Policy on Access to Research Outputs *(2007)	...CIHR has a fundamental interest in ensuring that the findings that result from the research it funds, including research publications and publication-related data, are available to the widest possible audience, and at the earliest possible opportunity
Genome Canada, *Data Release and Resource Sharing Policy *(2008)	Genome Canada is committed to the principle of rapid data release and sharing of unique resources to the scientific community; Genome Canada-funded projects must therefore share data and resources in a timely fashion with minimal or no restrictions. .... Genome Canada applicants must provide a Data and Resource Sharing Plan as part of their application
Genome Canada, *Policy on Access to Research Publications *(2008)	Research publications are an important output of the research funded by Genome Canada and free, online access to these publications is paramount. Genome Canada recommends that peer reviewed publications that have been supported, in whole or in part, by Genome Canada be made freely accessible online, in a central or institutional repository, as soon as possible, and, at the latest, six months after the publication date
National Institutes of Health (US), *Final Statement on Sharing Research Data *(2003)	...data sharing is essential for expedited translation of research results into knowledge, products, and procedures to improve human health. ... ...all investigator-initiated applications with direct costs greater than $500,000 in any single year will be expected to address data sharing in their application
Bill & Melinda Gates Foundation (US), *Global Health Data Access Principles *(2011)	Grantees will be required...to facilitate the prompt and broad dissemination of data. ... When possible, data should be deposited into public-access repositories
California Institute for Regenerative Medicine (CIRM; US), *Stem Cell Grant Regulations *(2006)	Grantees shall share biomedical materials first created under CIRM funding and described in published scientific articles for research purposes in California within 60 days of receipt of a request and without bias as to the affiliation of the requestor unless legally precluded. ...[S]uch materials are to be shared without cost or at the actual cost of providing the material without an allocation of costs for overhead, research, discovery or other non-direct costs of providing the material
Howard Hughes Medical Institute (HHMI; US), *Sharing of Publication-Related Materials, Data and Software *(2007)	...the mission of HHMI is to move biomedical science forward, and broad dissemination of research tools and reagents created by its investigators very much supports that mission...
Medical Research Council (UK), *Data Sharing Policy *(2011)	Research is a public good and, as such, data should be made available to the maximum extent possible, and should be shared in a timely and responsible manner
Biotechnology and Biomedical Sciences Research Council (UK), *Data Sharing Policy *(2010)	Members of the community are expected and encouraged to practise and promote data sharing, determine standards and best practice, and create a scientific culture in which data sharing is embedded. BBSRC will provide support and funding to facilitate this. ... BBSRC expects research data generated as a result of BBSRC support to be made available with as few restrictions as possible in a timely and responsible manner to the scientific community for subsequent research
Wellcome Trust (UK), *Policy on Data Management and Sharing *(2010)	Researchers should maximise the availability of data as the maximization of the value of research depends on fostering a culture in which both data-generators and data-users act with integrity and transparency in managing, using, and sharing data

### A policy conflict?

On the surface, these policy trends seem to create an untenable situation within the research community. We might reasonably ask if there is, in fact, an irreconcilable conflict? Can researchers reasonably be expected to embrace the commercialization and the 'open science' imperatives? And what is the impact of one upon the other?

Before briefly considering these questions, we should say a word about the two models. First, many are skeptical that the narrow commercialization agenda will work. As noted by Hopkins, *et al*. [64], '[t]he translation of advances in bioscience into new technology is far more difficult, costly and time-consuming than many policymakers believe.' There is evidence that approaches to commercialization, such as partnering with industry and the promotion of start-up companies, simply do not work [65]. Moreover, the early perception that patents, a common outcome of commercialization, could procure a substantial source of income for academic institutions has now been strongly refuted by the available evidence [34]. Biotechnology patents have also raised substantial ethical and scientific controversies and are facing increasing legal challenges (*Oliver Brustle *v. *Greenpeace e. V*. (2011), *Association for Molecular Pathology et al*. v. *USPTO et al*. (2010; overturned in part by the Court of Appeals for the Federal Circuit)). To be fair, open scientific collaborations in biotechnology are still in their infancy, and, like commercialization, have much to prove in terms of delivering economic, social, or clinical benefits [66], though there certainly have been anecdotal examples of open models succeeding [45,67]. One of the major challenges for proponents of such collaborative models will be to convince governments and industry of the value of this particular business model, and to obtain their participation in more open initiatives.

On the question of irreconcilability, more than a few scholars have suggested that the pressure on researchers to obtain IP rights, a by-product of the commercialization instruction, conflicts directly with the idea of free exchange of scientific knowledge [68]. While the data on the impact of patents is equivocal (there is, for example, little solid research that shows a clear 'anti-commons' effect [32,69,70], research gives teeth to the concern that commercialization pressure hurts open scientific collaboration [71]. One recent study concluded that industry sponsorship of university research 'jeopardizes public disclosure of academic research' [72]. Other studies have found that commercialization activity negatively impacts researcher collaboration [73]. This occurred despite researchers being part of a collaborative network. Given that collaboration is an important element of an open (scientific) culture, this research, which is buttressed by other recent work [74], supports the contention that there is, at a minimum, a serious policy tension. Indeed, it was concluded that, 'policies directed at enhancing collaborative networks and policies directed at commercialization are moderately antagonistic' [73]. This is a position shared by some of the TTO respondents in that study, who felt that (the dominant) commercialization pressure cut against the traditional mandate of universities, which, as one TTO official put it, was 'the discovery of new knowledge' [37].

Despite the above, we take a more circumspect view on the question of irreconcilability of commercialization, on the one hand, and open scientific collaboration, on the other. Certainly there is a tension! But we should not presume that this tension is necessarily (all) bad. Managing the coexisting policy mandates (and tension), and keeping everyone (reasonably) happy will be a major policy and practical challenge in the coming years, but it is arguable that the dual pressures creates a balance that, in the aggregate, could be good for both science and society. Moreover, it is possible that the two mandates do not always conflict or are not always necessarily irreconcilable [75-77]. Ideally, both commercialization and open scientific collaboration could be viewed as complementary strategies within a larger framework of activities aimed at getting the optimal social and economical value from university research that must be alternatively chosen based on prevailing circumstances [11].

Openness in genomics is being promoted by the same research funding agencies that have been mandated for the last three decades with promoting commercialization. This at least offers the possibility of a uniform (or joined-up) voice being found. Of course, the positive potential of this dualism relies on funding agencies and research institutions collaborating to avoid the uncoordinated super-position of one innovation model over the other. Again, a clear message must be forged by the distinct yet closely tied communities that are relevant to the scientific undertaking, namely the political and the funding community, the international non-governmental organizations, and the research communities. Funding and institutional policies, research guidelines, and project protocols must all be harmonized insofar as they must all recognize the dualism and permit those operating on the ground to choose the course (or courses) that most effectively satisfies the needs of the public.

Currently, the appearance and language of conflict together with the often unreflexive nature of the balancing of apparently diverging interests is a real problem, albeit one that deserves further empirical study. Researchers have little space to come to grips with how they might, in practice, embrace both imperatives. Bearing this in mind, important questions for further empirical research include: to what extent do researchers sense this policy tension? If they perceive it, what effect is it having on their behavior? And how should individual researchers go about determining how to complete a grant application section covering the expected societal or economic benefit of research? (Should they be 'conservative' and talk about short-term commercialization, job creation and patenting opportunities, or should they take a chance and discuss open and rapid dissemination, societal benefits, and the advancement of science?)

Empirical research around these and other questions will go a long way to answering the third question above, what is the impact of one policy on the other, and to forging a path toward joined up and nuanced strategies that are paralleled across sectors and jurisdictions. At the moment, as alluded to above, researchers simply do not have the requisite training to navigate the patchwork or come up with ways to effectively blend them.

## Summary

In some respects, this situation defies a conclusion. We need to collectively and collaboratively forge a middle path that uses the best elements of both policy trends: an integrated and flexible approach to innovation. To some extent, the conflict that appears to exist is more a function of the disorganized accumulation of seemingly contradictory broad political mandates than of a true policy incompatibility. Ultimately much more empirical research is needed: (1) to document practices and difficulties encountered by researchers trying to navigate innovations policies and their potentially conflicting demands; (2) to determine the extent to which commercialization policies and open science policies actually conflict (or could be made to conflict less); (3) to determine the degree to which the contemporary innovation ethos conflicts with existing norms of scientific inquiry; and (4) to determine if the policy tensions are, in aggregate, harmful (or could be construed to have a more positive impact on research).

It would seem that the current dilemma is part of a larger questioning on how best to integrate the various policy approaches to innovation into a single coherent and measurable framework that would encompass both open scientific collaboration and commercialization into the more global and flexible approach we envision. In the current transitional period, it is hoped that funding agencies will be able to develop clear guideposts to assist researchers in finding their way across this research conflict (that is, helping them to determine how best to answer questions in their grant applications).

## Abbreviations

BBSRC: Biotechnology and Biomedical Sciences Research Council; CIHR: Canadian Institutes of Health Research; IP: intellectual property; MRC: Medical Research Council; TTO: technology transfer office.

## Authors' information

TC is a Canada Research Chair in Health Law and Policy, a Professor in the Faculty of Law and School of Public Health, and Research Director of the Health Law and Science Policy Group, University of Alberta. SHEH is Lecturer in Regulation and Risk in the School of Law, Deputy Director of the J Kenyon Mason Institute in Medicine, Life Sciences and the Law, and Research Fellow at INNOGEN, ESRC Centre for Social and Economic Research on Innovation in Genomics, and SCRIPT, AHRC Centre for Research on Intellectual Property and Technology Law, all at the University of Edinburgh. YJ is Assistant Professor at the Centre of Genomics and Policy, McGill University.

## Competing interests

None of the authors have any competing interests. It should be noted, however, that both TC and YJ currently receive research funds from the Stem Cell Network, an entity mentioned in the article. They have also, in the recent past, received funds from Genome Canada.

## Authors' contributions

Authorship is listed alphabetically. All authors (Caulfied, Harmon and Joly) contributed equally to the preparation of this article and have read and approved the final manuscript for publication.
